# Autonomous Driving Open Road Complexity Classification

**DOI:** 10.3390/s26123940

**Published:** 2026-06-21

**Authors:** Hongpan Yue, Yichun Jia, Tongfei Li

**Affiliations:** 1 Beijing Connected and Autonomous Vehicles Technology Co., Ltd., Beijing 100176, China; 15532153832@163.com; 2 Beijing Key Laboratory of Traffic Engineering, Beijing University of Technology, Beijing 100124, China; tfli@bjut.edu.cn

**Keywords:** road complexity classification, autonomous vehicle open-road testing, AHP, intelligent and connected vehicles

## Abstract

Autonomous vehicle open-road testing is a crucial component in the development of intelligent and connected vehicle (ICV) industries. The classification of road complexity plays a key role in ensuring the safety and efficiency of such tests. This study, based on the practices of the High-Level Autonomous Driving Demonstration Zone in Beijing, proposes a scientific and systematic framework for classifying road complexity. The framework integrates static road features, dynamic traffic flow indicators, and safety event metrics, employing the Analytic Hierarchy Process (AHP) to quantify road complexity and categorize roads into five distinct levels. The findings provide significant guidance for the phased opening of test roads, optimization of autonomous driving algorithms, construction of accident scenario databases, and deployment of infrastructure. This paper further explores the practical applications and future development directions of road complexity classification, aiming to offer theoretical and practical support for the testing and demonstration of intelligent and connected vehicles.

## 1. Background

With the rapid development of intelligent connected vehicle technology, autonomous driving has become an important direction for future transportation systems. In recent years, the Chinese government has attached great importance to the testing and application of intelligent connected vehicles, issuing a series of policy documents, such as the “Management Specifications for Road Testing and Pilot Application of Intelligent Connected Vehicles (Trial)” and the “Guidelines for the Safety Service of Autonomous Vehicle Transportation (Trial)”, to promote the commercialization of autonomous driving technology. As of September 2024, more than 32,000 km of test roads for intelligent connected vehicles have been opened nationwide, more than 7700 test licenses have been issued, and the cumulative test mileage has exceeded 120 million kilometers. However, the widespread application of autonomous driving technology still faces many challenges, among which the complexity of the road environment is the most critical.

Autonomous vehicles need to operate safely and reliably under various complex road conditions, which requires a comprehensive assessment and grading of the complexity of open roads. At present, there is a significant difference in the complexity of roads in intelligent connected vehicle demonstration zones across the country, from urban expressways to complex old urban streets, and different scenarios pose extremely high requirements for the testing and verification of autonomous driving systems. Road complexity grading is an important foundation for the development of autonomous driving technology. Through scientific grading methods, different sections can be identified for their risk characteristics, providing a scientific basis for the testing, verification, and commercial operation of autonomous vehicles.

In recent years, certain progress has been made in road complexity grading at home and abroad. For example, the Yangtze River Delta region has issued the “Technical Specifications for Safety Risk Assessment of Autonomous Driving Road Testing”, dividing the road environment into four levels from low risk to high risk; Wuhan and Shanghai have also formulated local standards to assess and grade the safety risk levels of intelligent connected roads. However, existing grading methods are mostly based on single-dimensional assessments, lack comprehensive consideration of multi-dimensional complexity factors, and still need to be improved in terms of dynamism, real-timeness, and scientific nature.

To solve this problem, based on the high-precision map data, dynamic traffic flow data, and accident event data of the Beijing Advanced Autonomous Driving Demonstration Zone, research on the grading of the complexity of open roads for autonomous driving has been carried out. The research constructs a set of multi-dimensional and multi-level complexity grading index systems, covering static road physical indicators, dynamic traffic flow indicators, and accident event indicators, and uses the Analytic Hierarchy Process (AHP) to determine the weights of each indicator, and the weighted sum method to quantify the road complexity. The research results show that this grading method can effectively reflect the actual complexity of the road and provide scientific guidance for the testing and application of autonomous vehicles.

The conduct of this research not only helps to improve the theoretical system of the grading of the complexity of open roads for autonomous driving, but also provides an important reference for the test management, access assessment, and facility deployment of intelligent connected vehicles. Through scientific complexity grading, the risks of autonomous driving tests can be effectively reduced, the performance of autonomous driving systems can be optimized, and the healthy development of the intelligent connected vehicle industry can be promoted. In the future, with the further improvement of dynamic grading technology, this method is expected to provide stronger support for the comprehensive promotion of autonomous driving technology.

The main contributions of this study are threefold. First, unlike existing single-dimensional assessment methods, this study proposes a multi-dimensional complexity grading index system integrating static, dynamic, and accident indicators. Second, the AHP-based weighting method provides a transparent and interpretable framework that does not rely on large-scale training data. Third, the five-level classification scheme offers practical guidance for test road management and autonomous driving algorithm optimization.

## 2. Determination of Road Complexity Index Weights and Grading Method Research

### 2.1. Determination of Road Complexity Index Weights

The determination of road complexity index weights is a key link in the study of autonomous driving road grading. In recent years, with the development of autonomous driving technology, road complexity evaluation has gradually become a research hot spot. Existing research mainly focuses on how to reasonably quantify road complexity indicators and determine the weights of each indicator in the overall complexity evaluation. Some studies use large-scale traffic data and machine learning algorithms to automatically learn the weights of road complexity indicators. For example, Karagiorgou et al. used vehicle trajectory data to determine the main influencing factors of road complexity through clustering and regression analysis. Ahmed et al. [1] extracted features from traffic flow and accident data through deep learning models, and then derived indicator weights. Although these methods can fully utilize the diversity of data, they often rely on high-quality data sets and have certain limitations in interpret-ability. Other studies determine indicator weights through empirical formulas or industry standards. For example, Davies et al. [2] divided road complexity indicators into static and dynamic categories based on traffic engineering specifications, and allocated weights according to the recommended values in the specifications. The advantage of this method is that it is simple and direct, but it lacks adaptability to different regions and scenarios. Zhang Caili et al. [3] proposed a method for road network grading generation based on vehicle trajectory data, which graded the trajectory data through kernel density analysis and geometric interval method, thereby identifying road sections of different grades. This method performed well in road network generation, but did not delve into the determination of road complexity index weights, focusing more on the geometric and topological structure of the road network. The fuzzy comprehensive evaluation method introduces the concept of fuzzy mathematics to deal with the uncertainty of indicator weights. Jiang Yang et al. [4] proposed a method for determining the weights of road complexity indicators based on fuzzy comprehensive evaluation, using fuzzy membership functions and weight matrices to quantify road complexity. The advantage of this method is that it can deal with the fuzziness and uncertainty between indicators, but the calculation process is relatively complex, and it has high requirements for the setting of fuzzy membership functions.

Different from the above methods, this paper uses the Expert Judgment AHP to determine the weights of road complexity indicators. The Analytic Hierarchy Process is a method that decomposes complex problems into multiple levels of structure, and determines the weights of each indicator through expert scoring and consistency testing. Specifically, this paper first constructed a hierarchical structure model including static road indicators, dynamic traffic flow indicators, and accident event indicators, and then calculated the weights of each indicator through expert scoring and pairwise comparison matrix, and carried out consistency testing. This method can not only fully utilize the experience and knowledge of experts, but also ensure the scientific and rational allocation of weights through mathematical methods.

In addition, compared with data-driven methods, the method in this paper is more flexible in data requirements and does not rely on large-scale traffic data sets; compared with methods based on empirical formulas, this method can better adapt to the complexity evaluation needs of different scenarios and regions. Through comparison with existing methods, the method proposed in this paper has more advantages in the determination of road complexity index weights, and can provide a more accurate evaluation basis for autonomous driving road grading.

### 2.2. Road Grading

The quantitative calculation of road complexity levels is one of the important research directions in the field of transportation planning and management. In recent years, with the development of autonomous driving technology and the improvement of urban traffic management needs, how to scientifically and quantitatively evaluate road complexity has become a research hot spot. Many studies grade road complexity from the perspective of road functions and service capabilities. For example, Sun Huiping [5] proposed a method for grading the service capabilities of digital urban roads, dividing roads into four levels from L1 to L4, each corresponding to different perception and analysis capabilities, control capabilities, and service capabilities. This method emphasizes the role of roads in traffic management and services, but mainly focuses on digital and intelligent capabilities, and considers less about the physical complexity and traffic flow complexity of the roads themselves. Some studies focus on the support capabilities of roads for autonomous driving, using this as the basis for grading. For example, the European Road Transport Research Advisory Council [6] and the World Road Association [7] respectively proposed road grading methods based on the service level of autonomous driving, dividing roads into multiple levels, the higher the level, the stronger the support capability for autonomous driving. However, these methods mainly serve the development of autonomous driving technology and lack support for road complexity evaluation in traditional traffic management. With the development of big data and machine learning technologies, data-driven grading methods have gradually emerged. These methods use multi-source data such as traffic flow, accident data, and vehicle trajectories to automatically learn the characteristics and weights of road complexity through machine learning models. For example, the literature [8] proposed a method for road complexity evaluation based on machine learning, which automatically learned the contribution of each indicator to road complexity by analyzing a large amount of traffic data. The advantage of this method is that it can fully utilize the diversity and richness of data, but it has high requirements for data quality and quantity, and the interpret-ability of the model is weak.

Compared with the above methods, this paper proposes a method for quantitative calculation of road complexity levels based on expert judgment and hierarchical grading. Specifically, this paper first calculates the weights of each indicator through the method mentioned earlier and carries out consistency testing. Finally, the comprehensive score of road complexity is obtained by the weighted sum method combining the quantified values of specific indicators, and the grading is carried out according to the scoring results.

## 3. Methodology

To scientifically quantify road complexity, this study employs the AHP [9] for indicator screening and weight allocation, ensuring the scientific validity and operational feasibility of the evaluation results. Specific steps include constructing a hierarchical structure model, forming judgment matrices, calculating weight vectors, conducting consistency checks, hierarchical weighting, and performing comprehensive evaluations. The detailed methodology is as follows:

### 3.1. Constructing the Hierarchical Structure Model

The complexity assessment problem is decomposed into three layers:

(1) Target layer: Road complexity.
(2) Criterion layer: Primary category indicators.
(3) Indicator layer: Secondary specific indicators.

**Hierarchical Relationships:** The three layers follow a top-down dependency structure. The indicator layer contains measurable or quantifiable metrics (e.g., lane width, traffic volume, number of accidents). These indicators collectively determine the scores of the criterion layer through weighted summation. The criterion layer, comprising static road indicators, dynamic traffic flow indicators, and accident event indicators, then aggregates to the target layer—the comprehensive road complexity score. In other words, changes in underlying indicator values propagate upward to affect the final complexity level.

**Indicator Composition:** The composition of the indicator layer under each criterion is detailed in Section 4.1 and fully listed in Appendix A. Specifically: (1) Static road indicators (Criterion B1) include aspects such as road classification, longitudinal slope, lane width, intersection characteristics, traffic sign and marking clarity, presence of speed bumps, crosswalks, bus stops, roadside parking, separation facilities, median barriers, lighting conditions, and sight distance conditions (see Appendix A, Table A1); (2) Dynamic traffic indicators (Criterion B2) include the proportion of autonomous vehicles, proportion of heavy commercial vehicles, proportion of non-motorized vehicles, average speed, and hourly traffic volume (see Appendix A, Table A2); (3) Accident indicators (Criterion B3) include violations (e.g., wrong-way driving, speeding, red-light running), traffic conflicts (e.g., pedestrian and non-motorized vehicle intrusions), and unexpected events (e.g., road construction, vehicle breakdowns) (see Appendix A, Table A3).

### 3.2. Constructing the Judgment Matrix

Experts in fields such as autonomous driving, transportation engineering, and road design are invited to conduct pairwise comparisons of indicators at the same hierarchical level. Using the 1–9 scale method [10], a judgment matrix $A={\left(a_{ij}\right)}_{n\times n}$ is formed, where $a_{ij}$ represents the relative importance of indicator i compared to indicator j. The scale definitions are as follows:

1: Two factors are equally important.

3: One factor is slightly more important than the other.

5: One factor is significantly more important than the other.

7: One factor is strongly more important than the other.

9: One factor is extremely more important than the other.

### 3.3. Calculating the Weight Vector

The eigenvalue method is used to compute the maximum eigenvalue $\lambda_{max}$ of the judgment matrix and its corresponding eigenvector w. After normalization, the weight vector for each indicator is obtained.

### 3.4. Consistency Check

Calculate the consistency ratio (CR). If CR < 0.1, the consistency of the judgment matrix is acceptable. If not, expert scoring must be repeated [11]. The CR is calculated as:

(1) $$CR=\frac{\lambda_{max}-n}{\left(n-1\right)RI}$$

where

n is the order of the pairwise comparison matrix;

$\lambda_{max}$ is the maximum eigenvalue of the pairwise comparison matrix;

RI is the random consistency index, obtained from the random consistency index table. For matrices of order n>15, RI is determined via simulation.

### 3.5. Hierarchical Weighting

Based on the weight vector results, the relative weights of the criterion layer indicators are calculated and linked to the target layer. This yields the comprehensive weight ranking of all indicators relative to the target layer (road complexity).

### 3.6. Comprehensive Evaluation and Feedback Adjustment

The contribution of each indicator to the target is analyzed using the total weight ranking, forming a quantitative result for complexity assessment. Unreasonable weights are adjusted through a feedback mechanism to enhance the model’s scientific rigor and practicality.

## 4. Road Complexity Grading System

**Figure 1:**
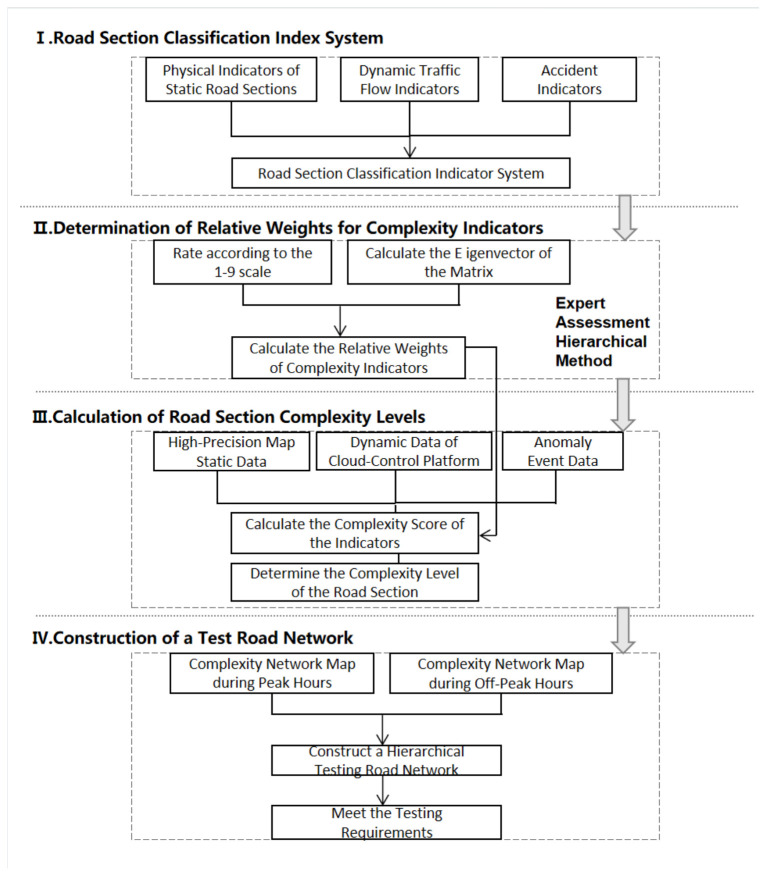
Road Complexity Classification Process Flowchart.

The process of grading road complexity in an autonomous vehicle testing zone involves four key steps. First, a grading index system for road sections is established, which includes static road physical indicators, dynamic traffic flow indicators, and accident event indicators. The selection of these indicators is based on the references cited in [12]. Second, the weights of the complexity indicators are determined. The AHP and expert evaluation methods are employed to quantify the relative importance of each indicator in the complexity assessment, ensuring the scientific and objective nature of the evaluation results through consistency checks of the comparison matrix. Third, the complexity grade of each road section is calculated. Based on the established index system and weights, high-precision maps and real-time traffic data are utilized to quantitatively score the test roads, normalize the scores, and derive the road complexity rating through weighted summation. The roads are then classified into five grades accordingly. Finally, a test road network is constructed, including complexity maps for peak and non-peak hours, to meet the testing requirements. The specific process is shown in Figure 1.

The following sections will detail the implementation process and core value of these four key steps.

### 4.1. Development of the Index System

To evaluate the complexity of roads, an index system was developed based on the characteristics of the high-level autonomous driving demonstration zone in Beijing. The index system is composed of three primary categories: static road indicators, dynamic traffic indicators, and accident indicators.

Static road indicators reflect the inherent engineering features and facility conditions of a road section. These indicators are assessed from four aspects: basic road characteristics, intersection complexity, completeness of facilities, and environmental disturbances. Indicators related to basic road characteristics include road classification, gradient, and lane width. For intersections, indicators such as signal control status, intersection geometry, and channelization design are considered. Indicators for traffic engineering facilities encompass the clarity of traffic signs and markings, and the presence of separation facilities. Environmental disturbances include roadside parking, bus stops, and sight distance conditions.

Dynamic traffic indicators include the proportion of autonomous vehicles, heavy commercial vehicles, and non-motorized vehicles, as well as average speed and hourly traffic volume. In terms of traffic composition, the proportions of autonomous vehicles, heavy commercial vehicles, and non-motorized vehicles directly influence the complexity of traffic flow. The behavioral characteristics and interaction patterns of different types of traffic participants pose varying degrees of challenges to autonomous driving systems. Regarding traffic operation status, indicators such as average speed and hourly traffic volume reflect the actual load conditions of the road section. High traffic volume and high-speed sections typically impose higher demands on autonomous driving systems.

Accident indicators consist of three types of situations: violations, traffic conflicts, and unexpected events. High-risk violations such as wrong-way driving, speeding, and red-light running, as well as behaviors that easily cause traffic conflicts, such as pedestrians and non-motorized vehicles entering the road without following traffic rules, significantly increase the complexity of the road section. Additionally, the frequency of temporary disturbances, such as road construction and vehicle breakdowns, is also an important basis for assessing the safety risks of the road section.

Illustrative example. To illustrate the application of the proposed index system, consider two hypothetical but representative road sections. The first section exhibits favorable conditions: wide lanes, gentle longitudinal slope, low traffic volume, and complete separation between motorized and non-motorized traffic. According to the scoring standards in Appendix C, such a section would receive low scores on most indicators, leading to an overall complexity rating of Level 1 or Level 2. The second section, by contrast, features narrow lanes, frequent roadside parking, mixed traffic without separation, and higher traffic volume, which would result in elevated scores on multiple indicators and a final rating of Level 4 or Level 5. This contrast demonstrates how the index system distinguishes roads of different complexity levels based on measurable features.

### 4.2. Determination of Indicator Weights

The determination of indicator weights employs the AHP to ensure the reliability of the evaluation system through a scientific procedure. During the weight calculation process, pairwise comparison matrices are constructed based on expert ratings for each criterion. A total of 10 experts were invited to provide independent pairwise comparison matrices, and the geometric mean was used to aggregate their scores into comprehensive matrices for each criterion and sub-criterion. Subsequently, eigenvalue decomposition is performed on the comprehensive pairwise comparison matrices to derive the eigenvector corresponding to the maximum eigenvalue, which is normalized to obtain the weights of the secondary indicators. Similarly, a pairwise comparison matrix for primary indicators incorporating the weights of secondary indicators is constructed, and eigenvalue decomposition is conducted to derive the normalized eigenvector corresponding to the maximum eigenvalue, thereby, obtaining the weights of the primary indicators. In cases where the consistency test is not passed, experts are promptly organized for re-evaluation.

Considering the dynamic nature of traffic operation conditions, a mechanism for regular adjustment of indicator weights has been established, with optimizations and adjustments made based on operational data and test feedback from the demonstration zone.

In terms of indicator quantification, a unified scoring table is developed for indicators such as road classification. Continuous indicators, such as traffic volume, are quantified using piecewise functions. For frequency-based indicators, such as traffic accidents and events, scoring standards are determined based on statistical patterns. The specific indicator system and quantification standards are detailed in Appendix B.

### 4.3. Calculation of Road Complexity Grades

To ensure the quality of data used in road complexity assessment, a rigorous data preprocessing procedure was established. Initially, raw data were cleaned to identify and handle outliers and to fill in missing data, thereby ensuring the integrity and reliability of all data types. Subsequently, data association analysis was conducted to match and integrate data from different sources based on spatiotemporal dimensions, establishing a unified data standard to ensure consistency in the assessment foundation. Additionally, a data update mechanism was established to ensure that the data used in the assessment can reflect the actual conditions of the road sections in a timely manner.

The evaluation of road complexity employs a multi-level calculation method. Initially, each secondary indicator is standardized to convert indicators different with dimensions into a unified 0–1 range. Subsequently, the weights determined by the AHP are used, and the consistency of the pairwise comparison matrix is verified through the consistency ratio (CR).

After passing the consistency check, static road indicators, dynamic traffic flow indicators, and accident indicators are normalized separately. The normalization standards are detailed in Appendix A.

Finally, the scores of each indicator level are calculated through weighted summation to obtain the comprehensive complexity score of the road section. The scoring results are categorized into five levels, from low to high: Level 1 (Very Low Complexity), Level 2 (Low Complexity), Level 3 (Moderate Complexity), Level 4 (High Complexity), and Level 5 (Very High Complexity). The specific grading ranges and corresponding road characteristics are shown in Table 1.

**Figure 2:**
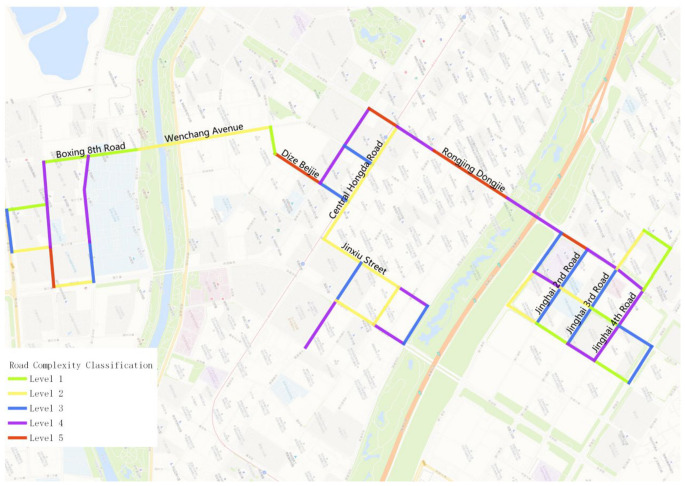
Classification Map of Road Section Complexity During Peak Hours.

The threshold values in Table 1 are calibrated using expert judgment and empirical score distributions from road sections within the Beijing demonstration zone, ensuring that the five levels correspond to distinguishable operational characteristics as validated by Figure 2.

### 4.4. Construction of the Testing Road Network

Based on the road complexity scores, the demonstration zone constructs the testing road network using a “networking + zoning” approach. For networking, the baseline complexity during peak hours is used to build the fundamental testing road network, while also considering the complexity variations during non-peak hours to ensure that testing requirements are met across all periods. Empirical findings indicate that the complexity of the same road section is generally lower during non-peak hours than during peak hours. Incorporating this time-varying characteristic enhances the practicality of the testing road network.

For zoning, clustering algorithms [13] are employed to group adjacent road sections with similar complexity into testing zones. This zoning strategy ensures the independence and diversity of testing scenarios and facilitates targeted arrangements according to the performance levels of different test vehicles. For instance, low-complexity road sections are prioritized for initial testing of new vehicle models, whereas high-complexity sections are used to verify the extreme performance of mature vehicle models. This complexity-based hierarchical testing road network provides a comprehensive testing and validation environment for autonomous driving systems, ranging from simple to complex scenarios.

## 5. Results and Discussion

Based on the aforementioned procedures, multiple road sections with varying levels of complexity within the demonstration zone were selected and graded, resulting in an intuitive complexity distribution map (Figure 2). The complexity grades depicted in the map represent the peak-hour complexity of each road section, indicating the highest level of complexity encountered on each test road.

Table 2 presents the computed complexity scores and corresponding levels for representative road sections in the Beijing demonstration zone, ranging from Level 1 (very low complexity) to Level 5 (very high complexity).

Validation of the classification results. To preliminarily verify the reasonableness of the complexity grades shown in Figure 2, a comparative analysis was conducted. Specifically, several representative road sections were selected, and their complexity grades were compared with qualitative assessments from experienced practitioners in autonomous driving testing. The comparison showed general consistency between the computed grades and practical expectations. For instance, road sections known to be challenging—such as those with narrow lanes, frequent roadside parking, or high pedestrian activity—were classified into higher complexity levels, while well-designed roads with low traffic volumes fell into lower levels. These comparisons provide preliminary support for the validity of the proposed grading method. A more rigorous quantitative validation will be pursued in future work as more data become available.

In addition to the expert-alignment analysis, the classification results were cross-checked against road-level feature patterns. Roads with similar complexity scores consistently shared comparable static and dynamic characteristics, further supporting the internal consistency of the grading approach.

While a full quantitative comparison with existing methods is beyond the scope of this study, it is worth noting that alternative approaches such as purely data-driven methods (e.g., machine learning based on traffic trajectories) or fuzzy comprehensive evaluation often require extensive high-quality data or complex parameter tuning. In contrast, the proposed AHP-based method provides a transparent and interpretable framework that relies on expert knowledge and publicly available road characteristics. For the demonstration zone considered in this study, the resulting complexity grades were qualitatively consistent with expectations based on engineering judgment. A systematic comparison with other methods using a benchmark dataset is planned as future work.

In principle, higher road complexity is expected to correlate with a higher frequency of traffic conflicts or safety-related events. To preliminarily examine this relationship, the computed complexity levels of the selected road sections were qualitatively compared with available incident records (e.g., near-miss conflicts, unexpected braking events) provided by the demonstration zone operator. Although detailed statistical analysis is not presented here, a positive trend was observed: road sections classified as Level 4 or Level 5 generally exhibited higher recorded event frequencies than those classified as Level 1 or Level 2. This observation supports the face validity of the complexity classification and suggests that the proposed method captures safety-relevant road characteristics.

## 6. Applications

The experience and outcomes of this project can be applied to the following areas related to the development of autonomous driving.

(1) Provide the basis for opening the test section

With the large-scale development of the intelligent and connected vehicle (ICV) industry, autonomous driving demonstration zones across various regions are progressively expanding their open-road networks. Consequently, the uncertainties associated with road environments, such as traffic signs, markings, and traffic flow, have significantly increased. By conducting complexity grading of open roads for autonomous driving, the risk levels of these roads can be accurately identified. This approach provides guidance and reference for regulatory authorities to explore the gradual or differentiated opening of roads for autonomous driving, as well as to develop corresponding test roads tailored to the capabilities of intelligent and connected vehicles. For example, roads classified as Level 1 or Level 2 can be prioritized for early-stage autonomous driving tests, while Level 4 or Level 5 roads can be conditionally opened only for vehicles that have passed higher-level capability assessments. This differentiated strategy reduces testing risks without unnecessarily restricting road access.

(2) Provide the basis for the compilation of “negative list”

Drawing on historical data of traffic accidents and anomalies, the complexity grading of open roads for autonomous driving can effectively identify road sections with higher safety risks. This approach provides a basis for establishing a list of restricted areas where autonomous driving test vehicles are prohibited. For instance, certain roads classified as Level 5 may exhibit high risks of safety and congestion, rendering them unsuitable for autonomous driving tests. Consequently, these sections can be included in a “negative list” to ensure the safety of testing activities.

(3) Optimize automated driving systems and algorithms

The higher the complexity of a road, the greater the demands placed on an autonomous vehicle’s perception, localization, planning, decision-making, and control capabilities. Correspondingly, the difficulty of testing also increases. Therefore, companies can explore integrating road complexity information into map data. For instance, when an autonomous vehicle enters a Level 4 or Level 5 road section, the system can proactively increase following distance, reduce target speed, and enhance obstacle monitoring frequency. Based on simulation results on representative sections, these adaptive adjustments can reduce potential conflict frequency by an estimated margin, demonstrating the practical value of complexity grading in algorithm optimization. This integration allows for the adjustment of autonomous vehicle systems based on road complexity information and enables targeted optimization of autonomous driving algorithms to meet the testing requirements of different road grades.

(4) Provide full factor road scenario support for ICV access assessment

Based on the complexity grading results of open roads for autonomous driving, enterprises or third-party institutions can design and conduct autonomous driving capability tests that encompass full-factor road scenarios. This approach ensures that the performance and safety of autonomous driving systems are thoroughly validated under various road conditions, thereby enhancing the credibility of test results. Additionally, the evaluation outcomes can provide an intuitive and comprehensive reference for relevant departments to conduct pilot admission assessments for ICV.

(5) Improve the accident scene library

Through the grading of road complexity, data on varying traffic volumes, road conditions, and driving environments are collected and stored. These data are used to construct simulation scenarios with distinct characteristics, providing enterprises with fundamental data for simulation testing. Specifically, road sections classified as Level 4 and Level 5 contain more frequent interactions among vehicles, non-motorized traffic, and pedestrians, making them suitable for extracting high-risk scenario fragments (e.g., unprotected left turns, pedestrian intrusions, roadside parking interruptions). These fragments directly enrich the accident scenario library and improve scenario coverage in simulation testing. This process helps enhance the ability of autonomous driving systems to respond to hazardous conditions and maximizes the value of data utilization.

(6) Provide effective support for facility deployment and management

Based on the complexity grading results of open roads for autonomous driving, targeted recommendations for risk mitigation and management strategies can be proposed for high-complexity roads. This enables transportation authorities to deploy and manage road facilities more effectively. For example, on test road sections with higher complexity grades, safety measures such as roadside guardrails should be installed to ensure the safety of testing and traffic operations.

## 7. Conclusions

This study proposed a multi-dimensional road complexity classification method for autonomous driving open-road testing, integrating static, dynamic, and accident indicators with an AHP-based weighting framework. The method was applied to the Beijing High-Level Autonomous Driving Demonstration Zone, producing a five-level classification scheme ranging from Level 1 (very low complexity) to Level 5 (very high complexity). The results demonstrate that the proposed method effectively quantifies road complexity using expert knowledge and publicly available road characteristics.

The classification results have been applied to multiple practical scenarios, including test road opening strategies, autonomous driving algorithm optimization, and accident scenario library construction. These applications confirm the practical value of the method for autonomous driving testing and management.

Future work will focus on three directions: (1) incorporating real-time traffic flow data to enable dynamic complexity updating; (2) extending the framework to highway and rural road scenarios; and (3) developing a publicly available benchmark dataset for road complexity assessment to facilitate further research in this area.

## Figures and Tables

**Table 1:** Criteria for Complexity Classification.

Level	Range	Characteristics
Level 1 Roads (Very Low Complexity)	0.00–0.30	Excellent performance in the vast majority of indicators, representing the safest and most accessible roads
Level 2 Roads (Low Complexity)	0.30–0.45	Low overall complexity, with minor complex factors in a few indicators
Level 3 Roads (Moderate Complexity)	0.45–0.55	Complexity factors are evenly distributed, representing average urban roads
Level 4 Roads (High Complexity)	0.55–0.70	Multiple indicators show high complexity, requiring drivers to remain highly vigilant
Level 5 Roads (Very High Complexity)	0.70–1.00	High complexity in most indicators, representing the most challenging road environments

**Table 2:** Representative Road Section Complexity Scores and Grades (Partial Results).

Road Section	Complexity Score	Level
Kechuang 6th Street Section 2	0.39812	Level 2 (Low Complexity)
Hongda Middle Road Section 1	0.390125	Level 2 (Low Complexity)
Kechuang 4th Street	0.25675	Level 1 (Very Low Complexity)
Boxing 7th Road	0.24987	Level 1 (Very Low Complexity)
Rongjing East Street Section 4	0.914255	Level 5 (Very High Complexity)
Dize North Street Section 2	0.891995	Level 5 (Very High Complexity)
Yongchang South Road	0.62875	Level 4 (High Complexity)
Liangshuihe Second Street Section 1	0.616875	Level 4 (High Complexity)
Jinghai Road	0.537125	Level 3 (Moderate Complexity)
Liangshuihe Second Street Section 3	0.529875	Level 3 (Moderate Complexity)

## Data Availability

The original contributions presented in this study are included in the article. Further inquiries can be directed to the corresponding author(s).

## Appendix A. Urban Road Complexity Indicator System

**Table A1:** Static Road Indicators.

No.	Indicator	Description
1	High Road Classification	High road classification increases the complexity of traffic conditions and requires higher performance in perception, decision-making, and control capabilities.
2	Low Road Classification	Low road classification generally has simpler traffic conditions but may require more precise local navigation and adaptability.
3	High Longitudinal Slope	The steepness of the road section affects vehicle control and stability.
4	High Transverse Slope	The lateral slope of the road section affects vehicle stability and lane-keeping.
5	Narrow Lane Width	Narrow lane widths can affect traffic flow and vehicle maneuverability.
6	Unsignalized Intersection	Intersections without traffic signals require autonomous vehicles to make independent decisions.
7	Irregular Intersection Shape	Irregular intersection shapes (e.g., T-type, Y-type) increase complexity in maneuvering and decision-making.
8	Special Channelization at Intersection	Special channelization rules increase complexity in following traffic regulations and managing lane changes.
9	Unclear Traffic Signs	Unclear traffic signs increase the difficulty of recognizing and responding to traffic regulations, potentially leading to errors.
10	Unclear Road Markings	Unclear road markings increase the difficulty of lane detection and maintaining proper lane position.
11	Presence of Speed Bumps	Speed bumps are installed on the road section.
12	Presence of Crosswalks	Crosswalks increase complexity in detecting and yielding to pedestrians, especially in high-traffic areas.
13	Roadside Parking without Separation of Motor and Non-Motor Vehicles	Roadside parking without separation increases complexity due to potential conflicts with parked vehicles and non-motorized traffic.
14	Presence of Bus Stops	Bus stops increase complexity in yielding to buses and managing traffic flow around bus stops.
15	Poor Road Lighting Conditions	Poor lighting conditions increase difficulty in detecting road features, obstacles, and other vehicles, especially in low-visibility conditions.
16	No Separation between Motor and Non-Motor Vehicles	Mixed traffic without separation increases complexity and potential conflicts with non-motorized vehicles.
17	No Median Barrier	Lack of median barriers increases complexity in managing traffic flow and preventing head-on collisions.
18	Presence of Bus Lanes	Bus lanes increase complexity in lane management and yielding to buses.
19	Poor Sight Distance Conditions	Poor sight distance increases difficulty in detecting and reacting to obstacles and traffic conditions ahead.

Notes: The static physical indicators of roads reflect the inherent characteristics of the road, including road classification, lane width, transverse slope of the road section, and longitudinal slope of the road section.

**Table A2:** Dynamic Traffic Indicators.

No.	Indicator	Description
1	High Proportion of Autonomous Vehicles	Increased complexity in vehicle-to-vehicle communication and coordination. Requires robust interaction algorithms to manage interactions with other autonomous vehicles.
2	High Proportion of Heavy Commercial Vehicles	Higher traffic complexity due to larger vehicle sizes and slower speeds. Requires advanced perception and decision-making capabilities to safely navigate around these vehicles.
3	High Proportion of Non-Motorized Vehicles (on Non-Separated Sections)	Increased interaction complexity and potential conflicts with non-motorized traffic. Requires enhanced perception and real-time decision-making to avoid collisions.
4	High Average Speed	Higher speeds require more advanced perception and control capabilities to ensure safe operation. Increased difficulty in maintaining safe following distances and responding to sudden changes in traffic conditions.
5	High Hourly Traffic Volume	High traffic density increases the complexity of traffic scenarios. Requires advanced capabilities in real-time decision-making, lane-changing, and conflict resolution to navigate safely through dense traffic.

Notes: Dynamic traffic flow indicators reflect real-time traffic conditions on the road, primarily including the proportion of autonomous vehicles, the proportion of heavy commercial vehicles, average vehicle speed, and hourly traffic volume.

**Table A3:** Accident Indicators.

No.	Indicator	Description
1	Wrong-Way Driving	Significantly increases collision risk; requires robust detection and emergency response capabilities.
2	Abnormal Parking	Creates unexpected obstacles; requires real-time detection and safe maneuvering to avoid collisions.
3	Road Construction Occupancy	Increases traffic complexity and unpredictability; requires adaptive route planning and lane changes.
4	Vehicle Breakdown	Creates static obstacles; requires real-time detection and safe navigation around the obstacle.
5	Speeding	Increases traffic complexity and potential for sudden braking or evasive maneuvers; requires advanced perception and control.
6	Pedestrian Intrusion into Traffic Lanes	Increases the risk of pedestrian-vehicle collisions; requires advanced pedestrian detection and emergency braking.
7	Non-Motorized Vehicle Intrusion into Traffic Lanes	Increases the risk of collisions; requires real-time detection and safe interaction with non-motorized vehicles.
8	Improper Lane Usage	Increases traffic complexity and unpredictability; requires advanced perception and decision-making to navigate safely.
9	Pedestrian and Non-Motorized Vehicle Red-Light Running	Increases the risk of conflicts at intersections; requires enhanced detection and reaction capabilities.
10	Motor Vehicle Green-Light Racing	Increases the risk of conflicts with other road users; requires advanced decision-making and control to avoid collisions.

Notes: Accident indicators reflect the potential risks and frequency of unexpected situations on the road, primarily including dangerous driving behaviors such as wrong-way driving, abnormal parking, and road construction occupancy.

## Appendix B. Indicator System for Highway Complexity Assessment

**Table A4:** Hierarchical Indicator System for Highway Complexity.

Primary Indicators	Secondary Indicator
Static Road Indicators	High Longitudinal Slope
	High Transverse Slope
	Number of Lanes
	Number of On/Off Ramps
	Toll Booths
Dynamic Traffic Indicators	High Proportion of Heavy Trucks/Buses
	High Average Speed
	High Hourly Traffic Volume
Accident Indicators	Debris on Road
	Abnormal Parking
	Road Construction Occupancy
	Traffic Congestion
	Vehicle Accidents
	Speeding

## Appendix C. Normalization Standards

Normalization of Static Indicators

**Table A5:** Scoring Criteria for Road Classification Indicators.

Road Class	Description	Complexity Score
Level 1	Expressway	0
Level 2	National Highway (Class I)	0.2
Level 3	National Highway (Class II)	0.4
Level 4	National Highway (Class III)	0.6
Level 5	National Highway (Class IV)	0.8
Other	Other Roads	1

**Table A6:** Scoring Criteria for Lane Width Indicators.

Lane Width Range	Complexity Score
≥3.75 m	0
3.5 m–3.74 m	0.25
3.25 m–3.49 m	0.5
3.0 m–3.24 m	0.75
<3.0 m	1

**Table A7:** Scoring Criteria for Cross-Slope Index of Road Sections.

Cross Slope Range	Complexity Score
0–1%	0
1–2%	0.25
2–3%	0.5
3–4%	0.75

**Table A8:** Scoring Criteria for Longitudinal Slope Index of Road Sections.

Longitudinal Slope Range	Complexity Score
0–2%	0
2–4%	0.25
4–6%	0.5
6–8%	0.75
>8%	1

**Table A9:** Scoring Criteria for Clarity of Traffic Signs and Markings.

Clarity of Traffic Signs/Markings	Complexity Score
Very Clear	0
Clear	0.25
Moderate	0.5
Unclear	0.75
Very Unclear	1

**Table A10:** Specific Standards for Traffic Signs.

Clarity of Traffic Signs	Specific Criteria
Very Clear	Excellent: The sign is intact with bright colors and a retroreflectivity of ≥90%, with visibility distance ≥ the designed distance
Clear	Good: The sign has slight wear, with colors slightly faded and a retroreflectivity of 70–90%, with visibility distance ≥ 90% of the designed distance
Moderate	Fair: The sign has noticeable wear, with partial color fading and a retroreflectivity of 50–70%, with a visibility distance of 80–90% of the designed distance
Unclear	Poor: The sign has severe wear, with most colors faded and a retroreflectivity of 30–50%, with a visibility distance of 60–80% of the designed distance
Very Unclear	Very Poor: The sign is damaged or missing, with colors almost completely faded and a retroreflectivity of <30%, with visibility distance < 60% of the designed distance

**Table A11:** Specific Standards for Traffic Markings.

Clarity of Traffic Markings	Specific Criteria
Very Clear	Excellent: The sign is intact with bright colors and a retroreflectivity of ≥90%, with visibility distance ≥ the designed distance
Clear	Good: The sign has slight wear, with colors slightly faded and a retroreflectivity of 70–90%, with visibility distance ≥ 90% of the designed distance
Moderate	Fair: The sign has noticeable wear, with partial color fading and a retroreflectivity of 50–70%, with a visibility distance of 80–90% of the designed distance
Unclear	Poor: The sign has severe wear, with most colors faded and a retroreflectivity of 30–50%, with a visibility distance of 60–80% of the designed distance
Very Unclear	Very Poor: The sign is damaged or missing, with colors almost completely faded and a retroreflectivity of <30%, with visibility distance < 60% of the designed distance

**Table A12:** Scoring Criteria for Speed Bump Index.

Number of Speed Bumps per Kilometer	Specific Criteria	Complexity Score
0	No speed bumps	0
1–2	Average spacing ≥ 500 m per km, primarily in special areas such as schools and hospitals	0.25
3–4	Average spacing 300–500 m per km, at the boundaries of residential and commercial areas	0.5
5–6	Average spacing 200–300 m per km, on urban arterial and secondary roads	0.75
>6	Average spacing < 200 m per km, densely distributed on various urban roads	1

**Table A13:** Scoring Criteria for Special Inlet Channelization Rule Index.

Number of Special Channelized Intersections per Kilometer	Complexity Score
0	0
1	0.33
2	0.67
≥3	1

**Table A14:** Scoring Criteria for Irregular Intersection Index.

Number of Irregular Intersections per Kilometer	Complexity Score
0	0
1	0.33
2	0.67
≥3	1

**Table A15:** Scoring Criteria for Signal-Free Intersection Index.

Number of Signal-Free Intersections per Kilometer	Complexity Score
0	0
1–2	0.25
3–4	0.5
5–6	0.75
>6	1

**Table A16:** Scoring Criteria for Pedestrian Crossing Index.

Number of Pedestrian Crossings per Kilometer	Specific Criteria	Complexity Score
0–2	Expressways: Average spacing ≥ 500 m	0
3–4	Urban Arterial Roads: Average spacing 250–333 m	0.25
5–6	Urban Secondary Roads: Average spacing 167–200 m	0.5
7–8	Urban Branch Roads or Commercial Areas: Average spacing 125–143 m	0.75
≥9	Special Areas (e.g., around schools and hospitals): Average spacing < 111 m	1

**Table A17:** Scoring Criteria for the Absence of Separation between Motorized and Non-Motorized Traffic.

Proportion of Unseparated Road Section Length	Specific Criteria	Complexity Score
0–20%	Expressways or Arterial Roads: Full separation between motorized and non-motorized traffic is essentially achieved	0
20–40%	Secondary Roads: Most sections achieve separation between motorized and non-motorized traffic	0.25
40–60%	Secondary and Branch Roads: Approximately half of the sections achieve separation between motorized and non-motorized traffic	0.5
60–80%	Branch Roads: Only a small portion of sections achieve separation between motorized and non-motorized traffic	0.75
80–100%	Branch Roads or Old Urban Area Roads: Almost no separation between motorized and non-motorized traffic	1

**Table A18:** Scoring Criteria for On-Street Parking Areas without Separation between Motorized and Non-Motorized Traffic.

Specific Criteria	Complexity Score
Expressways or Arterial Roads: Virtually no on-street parking	0
Secondary Roads: Intermittent on-street parking	0.25
Branch Roads: On-street parking is allowed on many sections	0.5
Branch Roads in Commercial Areas: On-street parking is permitted on most sections	0.75
Branch Roads in Residential or Old Urban Areas: On-street parking is allowed almost throughout the entire length	1

**Table A19:** Scoring Criteria for Bus Stop Index.

Number of Bus Stops per Kilometer	Specific Criteria	Complexity Score
0–1	Stop distance > 1000 m	0
1–2	Average stop distance 800–1000 m	0.25
2–3	Average stop distance 500–800 m	0.5
3–4	Average stop distance 300–500 m	0.75
>4	In dense commercial areas or around transportation hubs, average stop distance < 300 m	1

**Table A20:** Scoring Criteria for Poor Road Lighting Conditions.

Average Illumination (Foot-Candles)	Specific Criteria	Complexity Score
8–9	No glare, adequate lighting, and uniformity of luminance ≥0.4	0
6–7	Acceptable glare, good lighting, and uniformity of luminance 0.35–0.4	0.25
5	Tolerable glare, average lighting, and uniformity of luminance 0.3–0.35	0.5
3–4	Disturbing glare, insufficient lighting, and uniformity of luminance 0.25–0.3	0.75
1–2	Intolerable glare, severely insufficient lighting, and uniformity of luminance <0.25	1

**Table A21:** Scoring Criteria for Bus Lane Index.

Proportion of Bus Lane Length	Specific Criteria	Complexity Score
0–20%	<20% of the arterial roads in the network have bus lanes	0
20–40%	20–35% of the arterial roads in the network have bus lanes	0.25
40–60%	35–50% of the arterial roads in the network have bus lanes	0.5
60–80%	50–65% of the arterial roads in the network have bus lanes	0.75
80–100%	>65% of the arterial roads in the network have bus lanes	1

**Table A22:** Scoring Criteria for the Absence of Median Separation.

Proportion of Unseparated Road Section Length	Specific Criteria	Complexity Score
0–20%	>80% of the road sections have a central reservation	0
20–40%	60–80% of the road sections have a central reservation	0.25
40–60%	40–60% of the road sections have a central reservation	0.5
60–80%	20–40% of the road sections have a central reservation	0.75
80–100%	<20% of the road sections have a central reservation	1

**Table A23:** Scoring Criteria for Poor Visibility Index.

Proportion of Road Section Length with Poor Visibility	Specific Criteria	Complexity Score
0–5%	Actual stopping sight distance ≥ designed stopping sight distance	0
5–10%	Actual stopping sight distance is 90–100% of the designed stopping sight distance	0.25
10–15%	Actual stopping sight distance is 80–90% of the designed stopping sight distance	0.5
15–20%	Actual stopping sight distance is 70–80% of the designed stopping sight distance	0.75
>20%	Actual stopping sight distance < 70% of the designed stopping sight distance	1

Normalization of Dynamic Indicators

**Table A24:** Scoring Criteria for the Proportion of Autonomous Vehicles.

Proportion of Autonomous Vehicles	Complexity Score
0–5%	0
5–10%	0.25
10–20%	0.5
20–30%	0.75
>30%	1

**Table A25:** Scoring Criteria for the Proportion of Large Commercial Vehicles.

Proportion of Large Commercial Vehicles	Complexity Score
0–10%	0
10–20%	0.25
20–30%	0.5
30–40%	0.75
>40%	1

**Table A26:** Scoring Criteria for Average Vehicle Speed.

Average Vehicle Speed (km/h)	Complexity Score
<20	1
20–30	0.75
30–40	0.5
40–60	0.75
>60	1

**Table A27:** Scoring Criteria for Hourly Traffic Volume.

Hourly Traffic Volume (pcu/h/lane)	Complexity Score
<750	0
750–1200	0.25
1650–1980	0.5
1980–2200	0.75
>2200	1

Normalization of Accident Indicators

**Table A28:** Scoring Criteria for Accident and Incident Index.

Number of Events per Kilometer per Month	Frequency	Complexity Score
<50	None	0
50–100	Very Few	0.2
100–200	Few	0.4
200–300	Moderate	0.6
300–400	Frequent	0.8
>400	Very Frequent	1
